# The potential and challenges of TREM2-targeted therapy in Alzheimer’s disease: insights from the INVOKE-2 study

**DOI:** 10.3389/fnagi.2025.1576020

**Published:** 2025-04-25

**Authors:** Ya-nan Ma, Xiqi Hu, Kenji Karako, Peipei Song, Wei Tang, Ying Xia

**Affiliations:** ^1^Department of Neurosurgery, Haikou Affiliated Hospital of Central South University Xiangya School of Medicine, Haikou, China; ^2^Department of Surgery, Graduate School of Medicine, The University of Tokyo, Tokyo, Japan; ^3^National Center for Global Health and Medicine, Tokyo, Japan

**Keywords:** Alzheimer’s disease, TREM2, microglial, immune regulation, targeted therapy

## Abstract

Alzheimer’s disease (AD) is a severe neurodegenerative disorder with a growing global burden. With the rising incidence of AD, the need for novel therapeutic targets has become increasingly critical. TREM2, a receptor expressed on microglial cells, plays a crucial role in modulating neuroinflammation and clearing pathological substrates, making it a promising candidate for AD therapy. However, the recent clinical trial INVOKE-2 failed to demonstrate significant clinical benefits of the TREM2-targeted antibody AL002, raising doubts about the efficacy of TREM2-targeted methods. This article examines the role of TREM2 in AD pathogenesis, evaluates potential reasons for the disappointing outcomes of the INVOKE-2 trial, and discusses future directions for TREM2-based therapies. Factors such as treatment timing, dosage optimization, patient genetic variability, and combination therapy strategies are identified as critical determinants of therapeutic success. Future studies should aim to refine treatment strategies, identify precise indications, and explore the potential for combination therapies to enhance efficacy.

## 1 Introduction

Alzheimer’s Disease (AD) is a neurodegenerative disorder with severe social and economic impacts, and there are still no effective cures available. Its pathological features include amyloid plaques (Aβ), tau neurofibrillary tangles (NFTs), and pronounced neuroinflammatory responses (Ma et al., 2024). With the aging population, the incidence of AD continues to rise, necessitating the development of effective therapeutic strategies. However, most drug development efforts targeting Aβ or tau have not met expectations, suggesting the need to explore new therapeutic targets from the perspective of inflammation and immune regulation (Long and Holtzman, 2019).

In recent years, the brain’s inflammatory response and its primary regulatory cells-microglia-have garnered significant attention. Microglia are the resident immune cells of the central nervous system, and under pathological conditions, they contribute to the clearance of pathological Aβ, secrete inflammatory factors, and remodel neural networks (Hu et al., 2024). However, when their function becomes abnormal or their response is dysregulated, microglia can accelerate neuronal damage and disease progression. Therefore, modulating microglial function to alleviate neuroinflammation is considered a potential new direction for AD therapy.

Beyond Aβ and tau, the triggering receptor expressed on myeloid cells (TREM) family (TREM1, TREM2, TREM4, TREM5) has drawn attention for their role in innate immunity. TREM1 is known to amplify inflammation, whereas TREM2 is primarily involved in maintaining microglial homeostasis (Allcock et al., 2003; Mecca et al., 2018). TREM2, specifically expressed on microglia, is pivotal in Aβ clearance and immune regulation, making it a compelling therapeutic target in AD (Mecca et al., 2018). By binding to ligands, TREM2 activates downstream signaling pathways that regulate microglial activation and function (Ulland and Colonna, 2018). Genetic studies have shown that low-frequency variants of TREM2 are genetic risk factors for non-familial AD. This mutation leads to the substitution of arginine-47-histidine (R47H) in the extracellular immunoglobulin domain, which is significantly associated with an increased risk of AD. Individuals carrying this mutation have a 2–4 times higher risk of developing AD (Jonsson et al., 2013; Slattery et al., 2014). These findings provide the theoretical foundation for TREM2 as a potential therapeutic target for AD.

Based on this understanding, the development of TREM2-targeted drugs has gradually become a hot topic. TREM2 is believed to promote the clearance of Aβ, enhance neuroprotection, and reduce inflammation by activating microglia. Some studies have also shown that the activation of TREM2 can improve neuronal function and delay neurodegenerative changes (Fassler et al., 2023; Zhao et al., 2022). As a result, TREM2 pathway activators, such as AL002, have become promising therapeutic options (Long et al., 2024).

However, in the recent phase II INVOKE-2 clinical trial (NCT04592874), the TREM2-targeted antibody AL002 did not significantly slow the clinical progression of AD (Alector, 2024). No significant improvement was observed in brain amyloid levels in PET imaging or in fluid biomarkers. This result has raised questions about the actual role of TREM2 in AD treatment. This paper reviews the role of TREM2 in AD, analyzes the potential reasons why the INVOKE-2 trial did not meet expectations, and explores possible future directions for TREM2-targeted therapy. The goal is to provide insights into innovative therapeutic strategies for AD.

## 2 The mechanisms of TREM2 in AD

**FIGURE 1:**
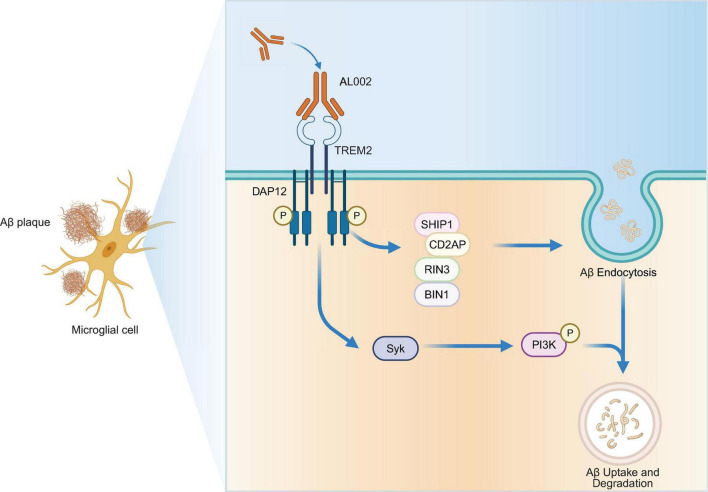
Mechanism of action of AL002, a TREM2-targeted agonistic antibody, in modulating microglial function and Aβ clearance. AL002 is a humanized monoclonal antibody that binds to TREM2 on the surface of microglia, promoting the formation and stabilization of the TREM2–DAP12 receptor complex. Upon ligand engagement, DAP12 undergoes phosphorylation on its immunoreceptor tyrosine-based activation motifs (ITAMs), recruiting and activating spleen tyrosine kinase (Syk). This initiates downstream signaling cascades, including activation of PI3K, which supports microglial survival, proliferation, and phagocytic capacity. Concurrently, TREM2 signaling facilitates the recruitment of adaptor proteins such as SHIP1, CD2AP, RIN3, and BIN1, enhancing Aβ endocytosis. The internalized Aβ is subsequently trafficked to lysosomal compartments for degradation. Collectively, AL002-mediated TREM2 activation promotes microglial clearance of Aβ plaques, contributing to neuroprotection in Alzheimer’s disease.

TREM2 is a transmembrane receptor that requires the adaptor protein DAP12 for signaling. This interaction is mediated through a conserved lysine residue (K186) in TREM2’s transmembrane domain, which forms a salt bridge with DAP12’s aspartic acid (D50) in its transmembrane region (Mecca et al., 2018). Upon ligand binding, DAP12’s immunoreceptor tyrosine-based activation motifs (ITAMs) undergo phosphorylation, recruiting spleen tyrosine kinase (SYK) and activating downstream pathways including the PI3K/Akt, promoting microglial survival and function (Figure 1). Microglia are the resident immune cells of the central nervous system and play a crucial role in sensing changes in the neural environment, clearing pathological products, and maintaining tissue homeostasis. In the pathological progression of AD, microglial function undergoes significant alterations, including responses to pathological Aβ, phagocytosis of pathological deposits, and the secretion of inflammatory cytokines (Thakur et al., 2023). TREM2 plays a key regulatory role in these processes (Wang et al., 2015). By activating microglia, TREM2 causes them to transition from a resting state to an activated state, enhancing their ability to clear pathological products while also regulating neuroinflammation to prevent secondary neuronal damage caused by excessive inflammatory responses (Park et al., 2024).

TREM2 participates in the pathological regulation of AD through multiple mechanisms. Firstly, it promotes microglial proliferation and survival. In the early stages of AD pathology, TREM2 activates the PI3K/Akt pathway downstream of the DAP12 signaling pathway, enhancing microglial survival and increasing their numbers to cope with neuroinflammation and pathological deposits (Figure 1; Sayed et al., 2021). TREM2 also enhances microglial phagocytic capacity, allowing them to more effectively clear pathological Aβ and other neurotoxic substances (Qin et al., 2021). Microglia lacking TREM2 show a significant reduction in their ability to clear Aβ deposits (Wang et al., 2020). Furthermore, TREM2 plays a role in lipid metabolism regulation. The clearance of Aβ involves extensive lipid metabolism, and TREM2 interacts with lipid-binding molecules such as apolipoprotein E (ApoE) to promote microglial adaptation to metabolic demands (Krasemann et al., 2017). This function is closely related to the ApoE genotype, further emphasizing the importance of TREM2 in AD. Finally, TREM2 can regulate microglial inflammatory responses, reducing the excessive release of pro-inflammatory cytokines, thereby balancing the neuroinflammatory response and protecting neurons from excessive inflammatory damage (Long et al., 2024).

The function of TREM2 exhibits dynamic changes at different stages of AD. In the early stages of the disease, TREM2 enhances microglial function, promoting the clearance of Aβ and slowing the pathological progression. However, in the later stages, the expression levels of TREM2 significantly decrease, and microglial function gradually declines, which may lead to uncontrolled inflammation and exacerbated neuronal damage (Jay et al., 2015; Jay et al., 2017; Wang et al., 2016). This dynamic change suggests that TREM2-targeted therapies should be tailored to the different stages of the disease.

Genetic studies further highlight the critical role of TREM2 in AD. Individuals carrying the R47H mutation have a significantly higher risk of developing AD (Stefansson et al., 2024). These mutations may impair the binding ability of TREM2 to its ligands or inhibit its signaling functions, leading to microglial dysfunction. These mutations not only affect the clearance of Aβ but also disrupt lipid metabolism and inflammatory regulation, accelerating the pathological progression of the disease (Basha et al., 2023). Therefore, genetic background may determine the severity of TREM2 dysfunction and influence the effectiveness of targeted therapies.

## 3 Insights from the INVOKE-2 study

INVOKE-2 was a randomized, double-blind, placebo-controlled, dose-ranging phase II clinical trial designed to evaluate the safety and efficacy of AL002, a TREM2-targeting monoclonal antibody, in patients with early-stage AD (Jackson et al., 2022). Participants were randomized to receive one of three intravenous dosing regimens of AL002 (15 mg/kg every 4 weeks, 40 mg/kg every 4 weeks, or 60 mg/kg every 4 weeks) or placebo (Jackson et al., 2022). The study was conducted across multiple centers in 11 countries and followed a common-close design, in which participants remained on their assigned regimen until the last enrolled patient completed 48 weeks of treatment, with the total study duration extending up to 96 weeks (Alector, 2024). Despite target engagement, AL002 failed to meet primary endpoints, showing no significant slowing in cognitive decline or amyloid PET reduction. Although levels of soluble TREM2 (sTREM2) were elevated—indicating pharmacodynamic engagement—the treatment failed to produce measurable clinical or biomarker benefits. In addition, adverse events included amyloid-related imaging abnormalities (ARIA), raising safety concerns, especially in APOE ε4 carriers (Alector, 2024). These results not only pose new challenges for TREM2-targeted therapies but also offer valuable insights that help reassess the potential and limitations of TREM2 in AD treatment.

Firstly, the INVOKE-2 trial enrolled patients in the early stage of AD, but it remains uncertain whether this stage represents the optimal therapeutic window for TREM2 activation (Jackson et al., 2022). The study underscores the critical importance of treatment timing in TREM2-targeted interventions. TREM2 plays dynamic roles throughout the disease course—early microglial activation facilitates the clearance of pathological Aβ, while in later stages, excessive or prolonged activation may aggravate neuroinflammation and neuronal damage (Qin et al., 2021). Intervening too early or too late may therefore limit the therapeutic efficacy of TREM2 modulation. Future studies should consider stratifying participants based on TREM2 activity levels or related biomarkers to more accurately identify those most likely to benefit from such therapies.

Secondly, dosage and treatment duration may be critical factors affecting efficacy. The lack of significant clinical effects in INVOKE-2 might be partially attributed to insufficient drug dosing or inadequate treatment duration to realize the full potential of TREM2 activation. TREM2 activation is a complex immune-regulatory process, and excessively high doses may lead to microglial overactivation and excessive inflammation, while too low a dose may fail to effectively trigger the amyloid clearance function. Moreover, microglia need time to gradually adapt to activation signals and transition into a state conducive to clearing pathological deposits. Therefore, future studies must explore the relationship between dosage and efficacy in greater detail and assess the long-term effects of treatment.

Thirdly, the INVOKE-2 study also raises concerns about the safety of TREM2-targeted therapies. Some patients in the trial exhibited ARIA, a side effect commonly observed with anti-amyloid antibody treatments. These adverse events were primarily seen in patients receiving AL002. It remains uncertain whether there is an association between ARIA and genetic backgrounds such as the presence of the ApoE ε4 allele or patient sex (Biechele et al., 2020; Doran and Sawyer, 2024). Additionally, the occurrence of ARIA could be linked to microglial overactivation or alterations in the blood-brain barrier function triggered by the treatment (Doran and Sawyer, 2024; Piazza et al., 2022). These safety concerns suggest that, when designing TREM2-targeted drugs, careful consideration should be given to dosage adjustments, genetic backgrounds, and strategies for combining treatments with other therapies to mitigate risks.

Moreover, the secondary endpoint results of INVOKE-2 further underscore the complexity of TREM2-targeted therapies. The study found no significant improvements in secondary clinical outcomes such as cognition, function, and behavior, which may reflect the fact that TREM2 does not act in isolation in AD. TREM2 interacts with other immune pathways, metabolic pathways, and neuroprotective mechanisms. Thus, a single-target approach may be insufficient to overcome the complex pathology of AD. This suggests that future therapeutic strategies might need to combine anti-amyloid antibodies, anti-tau therapies, or other neuroprotective agents to more comprehensively slow disease progression (Cummings et al., 2024).

It is also important to note that the assessment of biomarkers has deepened our understanding of TREM2-targeted therapies. In INVOKE-2, AL002 failed to significantly improve fluid biomarkers associated with neuroinflammation or reduce amyloid plaque levels in PET imaging. This suggests that while TREM2 can regulate microglial activity, activating TREM2 may not be sufficient to significantly alter the core pathology of AD. These findings imply that our understanding of TREM2’s mechanisms may still be incomplete, and further research is needed to optimize therapeutic designs. Additionally, the selection of biomarkers may need to be more precise to capture the specific biological effects of TREM2 activation.

## 4 Future perspectives on TREM2-targeted therapy

Although the INVOKE-2 trial did not achieve its primary clinical endpoints, it provide valuable insights into the therapeutic landscape of TREM2-targeted interventions in AD. Rather than undermining the therapeutic rationale, the results highlight the biological complexity of microglial signaling and the need for a more refined and individualized approach to therapy.

The function of TREM2 is modulated by a variety of factors, including genetic background, disease stage, and individual biomarkers. While the R47H variant is well characterized in terms of impaired ligand binding and increased AD risk, other rare variants such as H157Y (Qiao et al., 2023), R62H (Kiianitsa et al., 2024), L211P (Jin et al., 2015), R136Q (Piccio et al., 2016), T96K (Piccio et al., 2016), T66M (Piccio et al., 2016), D87N (Guerreiro et al., 2013), and Q33X (Filipello et al., 2023) have also been identified. Given that TREM2 mutations can alter protein synthesis, maturation, and cleavage, the levels of sTREM2 in the extracellular space may be modulated by TREM2 genotype (Piccio et al., 2016). Therefore, genetic stratification will be essential for future trials to better identify populations likely to benefit from TREM2-targeted therapy.

In addition to genetic factors, the integration of fluid and imaging biomarkers into trial design will be critical for evaluating TREM2 pathway engagement and monitoring therapeutic outcomes. sTREM2, a marker of microglial activation, has emerged as a potential dynamic indicator of treatment response (Bieger et al., 2023; Warmenhoven et al., 2024). Biomarkers such as YKL-40 and glial fibrillary acidic protein (GFAP), measured in cerebrospinal fluid or plasma, are associated with glial activity and cognitive decline in preclinical AD (Warmenhoven et al., 2024). Therefore, the development of biomarkers that can specifically reflect microglial function and real-time TREM2 signaling activity is urgently needed to enable therapeutic monitoring and individualized dose adjustment.

Given the multifactorial etiology of AD, monotherapy targeting a single immune receptor may be insufficient. Combination strategies that integrate TREM2 agonists with anti-Aβ, anti-tau, or neuroimmune modulators may offer synergistic benefits (Fassler et al., 2021). In addition, antibody engineering approaches that improve blood-brain barrier penetration and optimize receptor clustering, may improve therapeutic effects (van Lengerich et al., 2023).

To enhance the therapeutic efficacy of TREM2-targeted interventions, clinical trial design should be further optimized to reflect the heterogeneity of AD and the dynamic activation states of microglia. Stratification frameworks incorporating TREM2 genotypes, sTREM2 levels, and transcriptional or functional markers of microglial states may facilitate the identification of patients most likely to respond to treatment. Moreover, dosing strategies should be adaptable, guided by real-time biomarker profiles, and optimized in accordance with biologically responsive phases of disease progression. Furthermore, traditional clinical endpoints may fail to comprehensively capture the dynamic roles of microglia and their impact on disease progression. Rather than focusing solely on changes in cognitive scores, it may be more beneficial to incorporate other secondary endpoints, such as brain imaging biomarkers, inflammatory markers, and patients’ quality of life (Shi et al., 2024).

## 5 Conclusion

The complexity and multifactorial pathological characteristics of AD make its treatment a major challenge in the field of neuroscience. TREM2, as a key regulatory molecule of microglial function, is believed to play an important role in the inflammatory response and the clearance of pathological proteins in AD. The INVOKE-2 study evaluated the efficacy of the TREM2-targeting antibody AL002 in early-stage AD patients. Although the results did not meet expectations, the scientific questions and therapeutic directions revealed by the trial offer important insights for the field of AD research. TREM2-targeted therapies may achieve greater clinical value in the future through precise stratification and combination therapies. Despite the challenges faced in current research, the potential of TREM2 as a therapeutic target in AD should not be overlooked. It represents a novel treatment mechanism, which aims to alleviate the pathological burden by modulating the innate immune function of the central nervous system.

In summary, TREM2-targeted therapy remains a promising and innovative direction in AD treatment, with significant room for exploration. By optimizing treatment strategies, integrating multidimensional research data, and developing new therapeutic approaches, TREM2-targeted drugs hold the potential to break through the current therapeutic dilemmas in AD and provide more effective treatment options for patients.
